# Participation in an Intensive Longitudinal Study with Weekly Web Surveys Over 2.5 Years

**DOI:** 10.2196/jmir.5422

**Published:** 2016-06-23

**Authors:** Jennifer Barber, Yasamin Kusunoki, Heather Gatny, Paul Schulz

**Affiliations:** ^1^ Institute for Social Research Population Studies Center University of Michigan Ann Arbor, MI United States; ^2^ Institute for Social Research Survey Research Center University of Michigan Ann Arbor, MI United States

**Keywords:** RDSL, web survey, longitudinal study, panel study, intensive data collection

## Abstract

**Background:**

Technological advances have made it easier for researchers to collect more frequent longitudinal data from survey respondents via personal computers, smartphones, and other mobile devices. Although technology has led to an increase in data-intensive longitudinal studies, little is known about attrition from such studies or the differences between respondents who complete frequently administered surveys in a timely manner, and respondents who do not.

**Objective:**

We examined respondent characteristics and behaviors associated with continued and on-time participation in a population-based intensive longitudinal study, using weekly web-based survey interviews over an extended period.

**Methods:**

We analyzed data from the Relationship Dynamics and Social Life study, an intensive longitudinal study that collected weekly web-based survey interviews for 2.5 years from 1003 18- and 19-year-olds to investigate factors shaping the dynamics of their sexual behavior, contraceptive use, and pregnancies.

**Results:**

Ordinary least squares and logistic regression analyses showed background respondent characteristics measured at baseline were associated with the number of days respondents remained enrolled in the study, the number of interviews they completed, and the odds that they were late completing interviews. In addition, we found that changes in pregnancy-related behaviors reported in the weekly interviews were associated with late completion of interviews. Specifically, after controlling for sociodemographic, personality, contact information, and prior experience variables, we found that weekly reports such as starting to have sex (odds ratio [OR] 1.17, 95% CI 1.03-1.32, *P*=.01), getting a new partner (OR 1.76, 95% CI 1.53-2.03, *P*<.001), stopping the use of contraception (OR 1.28, 95% CI 1.10-1.49, *P*=.001), and having a new pregnancy (OR 5.57, 95% CI 4.26-7.29, *P*<.001) were significantly associated with late survey completion. However, young women who reported changes in pregnancy-related behaviors also had lower levels of study attrition, and completed more interviews overall, than did their counterparts.

**Conclusions:**

We found that measures of participation in a longitudinal study with weekly web surveys varied not only by respondent characteristics, but also by behaviors measured across the surveys. Our analyses suggest that respondents who experience the behaviors measured by the study may maintain higher participation levels than respondents who do not experience those behaviors.

## Introduction

Intensive longitudinal data are measures collected from participants across multiple observations. The number of observations that make data *intensive* is subject to debate, and “may be in the tens, hundreds, or thousands” [1]. The first intensive longitudinal data collections used pagers (beepers) to collect timed or event-driven data [2]. These studies were based on small, non-representative samples (eg, 75 high school students). Today, the widespread use of personal computers, smartphones, and other mobile devices greatly facilitate intensive data collection on larger and more diverse samples [1,3,4]. As these technologies become less expensive and more pervasive, intensive longitudinal data collection is likely to become even more common in survey research [5].

One obvious risk for studies with frequent measurement is the potential that some respondents will feel burdened by the task and will not continue in the study, or will respond sporadically to measurement attempts. If these respondents are different from those who remain in the study or who participate more consistently, then bias becomes a threat to the study [6]. Much research has addressed the causes and consequences of continued participation in multi-wave studies, as attrition affects the extent to which the sample remaining at the end of the study represents the initial sampling frame [7-14]. Little research, however, has addressed attrition in a population-based *intensive* longitudinal study with weekly interviews over a multi-year period.

Analyses of respondent characteristics and behaviors affecting on-time participation, an issue that is especially crucial to longitudinal designs with frequent measurement, are also lacking. Computer-based interviewing methods may tailor survey questions to reflect late or skipped participation by referring to the period since the respondent’s last interview. However, at some point, late participation in frequent data collections weakens the data set. For example, a weekly survey in which each survey is completed four weeks late becomes a monthly survey. Little is known about the differences between respondents who complete frequent surveys in a timely manner and respondents who do not.

In this analysis, we examined factors associated with continued and on-time participation in the Relationship Dynamics and Social Life (RDSL) study. The RDSL study collected *weekly* data from 18- and 19-year-old women on their attitudes, relationships, sexual behaviors, contraceptive use, and pregnancy status for 2.5 years, amounting to 130 observations from each fully participating respondent by the end of the study. By asking respondents to recall events over just the prior week, the study greatly increased researchers’ ability to determine the sequences of events leading to pregnancy, and to identify reciprocal relationships among attitudes and behaviors.

Using the RDSL data, we analyzed variance by individual characteristics and behaviors in the number of days respondents remained enrolled in the study, the number of survey interviews they completed, and the odds that they were late completing survey interviews. The individual variables were obtained from respondents’ self-reported characteristics at baseline, and subsequent pregnancy-related behaviors and experiences were reported during the course of the study.

## Methods

### The Relationship Dynamics and Social Life Study

The RDSL used a population-based sample of 1003 respondents randomly selected from a list of 18- and 19-year-old women registered as having either a driver’s license or a Personal Identification Card (PID) in a single county in Michigan, USA. The sampling method was a cost-based decision; due to the relatively sparse distribution of 18- to 19-year-olds in the general population, random selection from this pool allowed us to avoid screening a large number of households. Comparison of the driver’s license and PID data by zip code to 2000 census-based population projections revealed 96% agreement between the frame count and the projections for this population (authors’ calculations).

A 60-minute face-to-face baseline survey interview was conducted between March 2008 and July 2009 to assess important aspects of family background, demographics, attitudes, romantic relationships, education, and career trajectories. At the conclusion of this baseline interview, respondents were invited to participate in a *journal* that was a structured survey interview every week for 2.5 years, focused on measures of pregnancy desires, contraceptive use, pregnancy, and relationship characteristics/behaviors such as commitment and sex. Respondents could elect to complete journal interviews on the web or with an interviewer via phone. Ninety-two percent of women who agreed to complete the journal had internet access and were encouraged to complete their journal surveys by web. The journal portion of the study concluded in January 2012.

**Figure 1:**
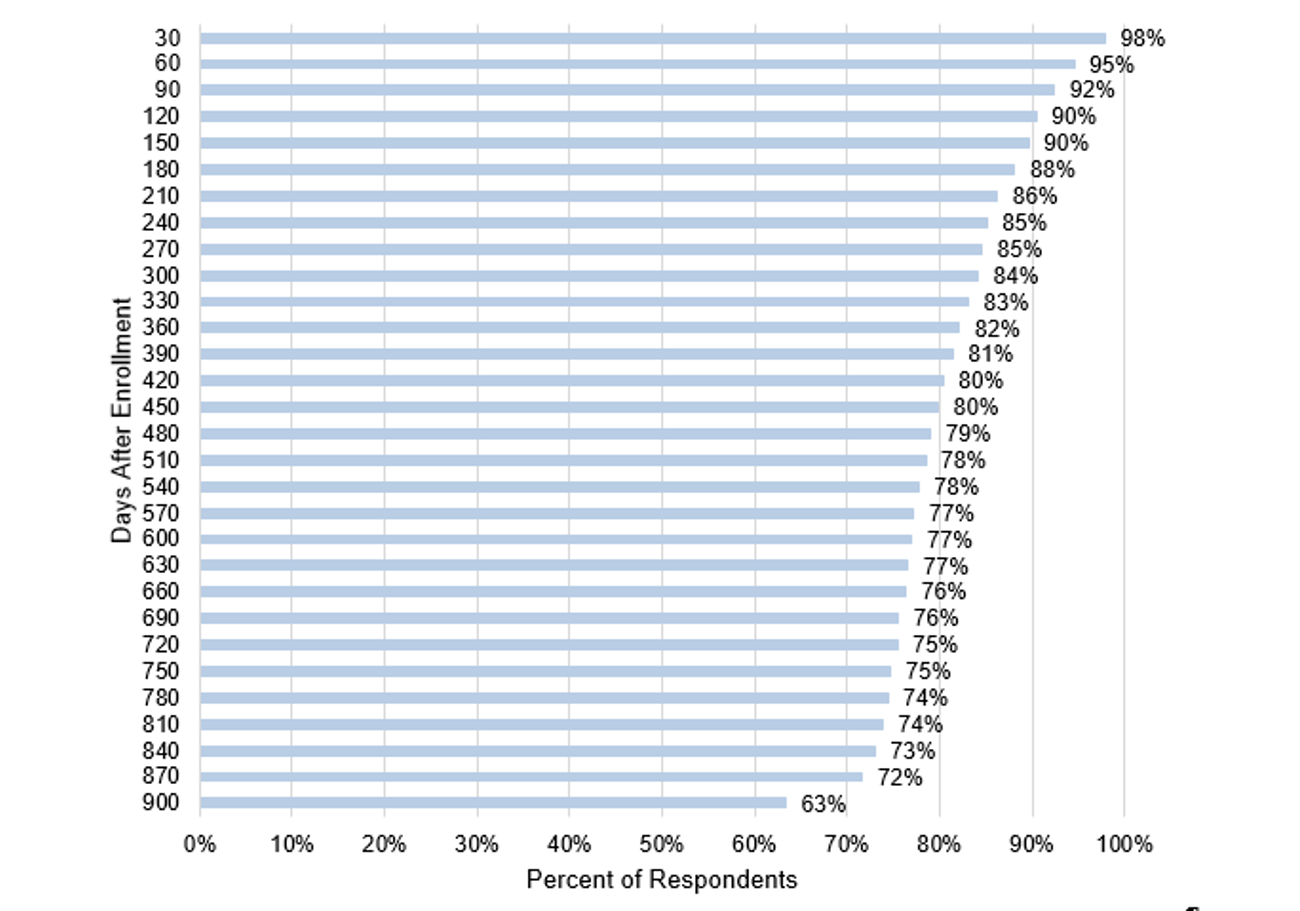
Continued participation in the journal.

The response rate for the baseline survey was 83.72% (1003/1198) [15]. Almost all of those who completed the baseline survey enrolled in the journal portion of the study (98.9%, 992/1003). The first journal was completed with the help of the professional interviewer immediately following the baseline interview. Of the 992 respondents who enrolled, 953 (96.1%) completed a second journal on their own. Of these 953, 741 (77.8%) participated in the journal interviews for at least 18 months and 604 (63.4%) completed their final interview at 900 or more days after enrollment (2.47 years). Figure 1 shows the percentage of respondents who remained enrolled in the study from 30 days after enrollment to 900 or more days after enrollment. Of the 128,960 possible weekly journals the study aimed to collect across 2.5 years from the 992 enrolled respondents, 58,594 (45.44%) were completed. Considering weekly survey questions were adjusted to refer to the period since the last interview for up to two weeks between surveys, this resulted in only a modest number of missing weeks. Item-specific missing data in the weekly interviews was also quite low at 3%.

RDSL researchers took several steps to minimize attrition and nonresponse in the study. First, we provided three types of participant incentives: money, tokens of appreciation, and regular reports on our study findings. Respondents were paid $1 per weekly journal with $5 bonuses for on-time completion of five weekly journals in a row. Journal incentives were distributed via reloadable, prepaid debit cards. Our prior research suggests that the ease and speed of the journal incentive payments encouraged continued participation [16]. Respondents also received small tokens of appreciation for their continued participation in the journal study (eg, a pen, compact, lip balm). We also provided regular reports to participants on current study findings, in large part because our previous ethnographic work with 18-year-olds indicated their desire to be kept apprised on the research to which they were contributing.

**Figure 2:**
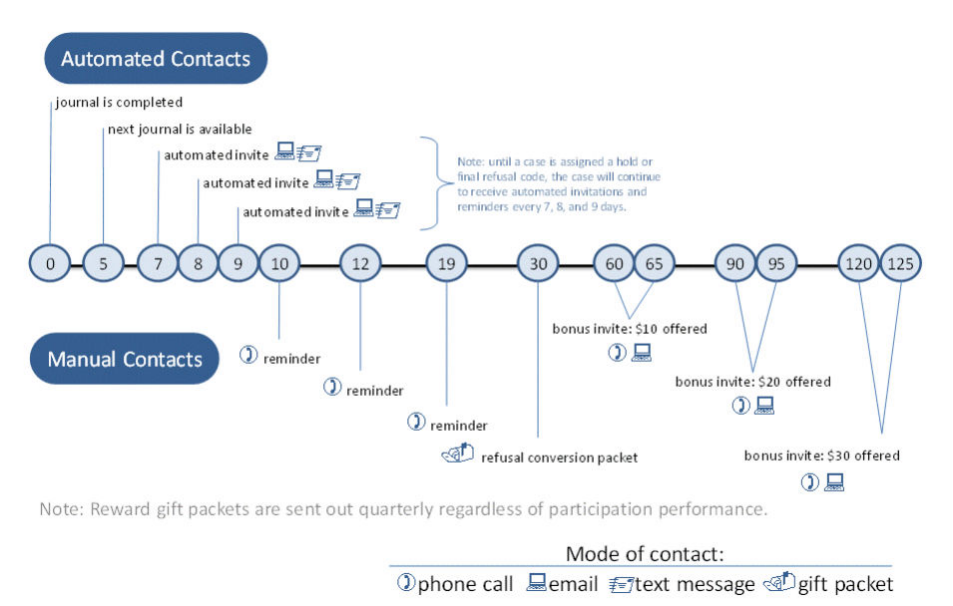
Heuristic of reminder protocol.

Second, we developed a multi-modal system of contacts to motivate and remind respondents to complete their weekly journals. The system, summarized in Figure 2, includes manual and automatic participant contact points that are recurrent and varied by mode, to increase response rates [17-20]. The day a journal is completed is day 0, with the next journal made available on the study’s website five days later, on day 5. On day 7, respondents were sent an automated invitation to complete the next journal via their selected contact mode (text messaging, email, or both). These automated invitations recurred on days 8 and 9, and on day 10 the reminder mode was switched to a telephone call. A second reminder call was made on day 12 and, if the journal was still not completed, a new automated invitation was sent via email or text message on day 14. This pattern of invitations and reminders was repeated until the respondent completed another journal interview or explicitly asked to be removed from the study. For participants not responding by day 30, we supplemented the automated email and text message reminders by mailing *refusal-conversion packets* to their homes that included a letter and a small gift (eg, pen, compact, lip balm). In addition, we offered respondents cash bonuses by phone/email for completing the next journal of $10 at day 60, $20 at day 90, and $30 at day 120.

Finally, to decrease respondent burden, we kept the survey short (approximately five minutes) and included only questions that were essential to the study (determinants of pregnancy). For instance, we did not include questions on finances, which respondents tend not to want to answer [11,21]. See Barber et al. (2011) for more information regarding the design and implementation of the RDSL study [22]. Sample journal interviews are provided in Multimedia Appendix 1.

### Measures

#### Non-Time-Varying Individual Characteristics

For the 953 RDSL study respondents who completed at least two journal interviews, Multimedia Appendix 2 shows mean measures for five sets of individual-level variables collected during the baseline survey and journal: (1) sociodemographic characteristics, (2) personality characteristics, (3) contact information/mode, (4) adolescent experiences (prior to the study) related to pregnancy, and (5) changes summarized over the study period. We explored the relationship of these non-dynamic variables to weekly survey participation.

##### Sociodemographic Characteristics

Previous research has documented that respondents from low socioeconomic backgrounds, and minority respondents, are more likely to drop out of panel studies [23,24]. In these analyses, we investigated a large set of sociodemographic characteristics we have included in other studies [25-31]. As shown in Multimedia Appendix 2, approximately one-third of the analytic sample was African American. The very few Latinas in the sample were coded according to their answer to the question of their race (some selected African American, while others selected white). To measure education of these 18- and 19-year-olds, we used a categorical variable combining enrollment and attainment: 13.5% (129/953) were enrolled in high school, 28.5% (272/953) were enrolled in a 2-year college/vocational program, 27.7% (264/953) were enrolled in 4-year college, 21.9% (209/953) had completed high school but were not enrolled in further education, and 8.3% (79/953) dropped out of high school and were not currently attending school. More than one-quarter (26.3%, 251/953) of the respondents were receiving public assistance. On average, respondents rated the importance of religion in their lives as 2.69 on a scale of 1 (not important) to 4 (more important than anything else). Over one-half (52.4%, 499/953) grew up with two parents, 39.8% (379/953) with one biological parent only (no step-parent), and 7.9% (75/953) in another arrangement (eg, with grandparents or an aunt). Respondents were relatively equally distributed across the four parental income categories, with the highest proportion (27.9%, 266/953) in the $15,000 - $44,999 category and 20.1% (192/953) not knowing their parents’ income. The average age at baseline was 19.19 years, with a median age of 19.

##### Personality Characteristics

Previous research has also documented the relationship between personality characteristics and study attrition. Attrition tends to be low among respondents who are assessed to be agreeable and/or conscientious, and higher among respondents who are extraverted [32]. The baseline interview included a series of questions adapted from the Neuroticism, Extraversion, and Openness to Experience-Five Factor Inventory (NEO-FFI), which measures extraversion, agreeableness, conscientiousness, neuroticism, and intellect/imagination [32]. Of the NEO-FFI’s 60 questions regarding personality traits, we included only the 40 used in Add Health Wave IV, presenting them as statements with five possible answers coded from 1 (strongly disagree) to 5 (strongly agree). As shown in Multimedia Appendix 2, respondents were distributed fairly evenly across the five personality categories using this coding scheme, with agreeableness and neuroticism ranking the highest and lowest in terms of respondents’ identification with the characteristic.

##### Contact Information/Mode

We also examined whether contact information provided, mode of the reminder, and mode of the journal interview were related to survey participation. Respondents were asked during the first journal interview (completed with the professional interviewer immediately following the baseline interview) to provide a home phone number, cell phone number, and an email address, with 84.1% (801/953) providing both an email address and a phone number and 15.9% (152/953) providing only one or neither. Respondents also selected their preferred automated reminder method for future journals, with 33.2% (316/953) electing to receive both a text and an email, and 66.8% (637/953) choosing one or neither reminder method. In addition, each week of the survey, respondents could elect to complete their interview via phone or web, with respondents ultimately completing 11.77% (6778/57,602) of all interviews by phone. Many of these phone call completions resulted from an interviewer-initiated call made on day 10 (the first day of manual contacts) in an attempt to complete the interview by phone. Thus, interviews completed by phone had a higher fraction late (1341/6778, 19.78%) than those completed on the web (5035/50,824, 9.91%).

##### Adolescent Experiences Related to Pregnancy

Although previous research has not linked pregnancy-related experiences to study attrition, we explored several salient experiences that occurred *prior to the study period*. At the baseline interview, 51.5% (491/953) of respondents reported having had sex by age 16, 59.7% (569/953) reported two or more sexual partners prior to the study period, 48.1% (458/953) reported having had sex without contraception, and 26.0% (248/953) reported having a prior pregnancy (17.0% [162/953] had one prior pregnancy and 9.0% [86/953] had two or more).

##### Summary of Changes During the Study Period

The bottom of Multimedia Appendix 2 includes three variables that summarize reported experiential changes over the course of the entire study: respondents reported an average of 1.83 new sexual partners, 53.9% (514/953) reported having sex without contraception at least once during the study, and 20.6% (196/953) reported a pregnancy during the study. We included these summary measures in our models presented in Multimedia Appendix 3 that used the 953 respondents as the units of analysis. Recall that we lost 39 of the original 992 respondents who only completed the first journal, and for whom we have no measures of change reported in the journal.

#### Time-Varying Pregnancy-Related Behaviors

We also explored the relationship of dynamic variables collected during the journal interviews to weekly survey participation. Table 1 provides means for the weekly interview questions about sex, partners, contraception, and pregnancy from the 57,602 journal interviews completed by the 953 respondents. Each question referred to the period since the prior journal (unless the interview was >14 days late, in which case it referred only to the prior week). As shown, most interviews indicated no changes from week to week, however, the following transitions were reported over the course of the study.

**Table 1:** Descriptive statistics for changes since prior journal (N=57,602 journals).

	Proportion/Mean
**Sex**	
No change	.84
Stopped having sex	.08
Started having sex	.08
**Partner transitions**	
No change	.90
Break-up (partner at time 1; no partner at time 2)	.04
New partner (no partner at time 1; partner at time 2)	.04
Partner switch (partner at time 1; different partner at time 2)	.03
**Contraceptive Use**	
No change	.92
Stopped using contraception	.04
Started using contraception	.04
**Pregnancy**	
No change	.99
Pregnancy ended	.005
New pregnancy	.004

An average of 7.65% (4408/57,602) of all journal interviews indicated that the respondent had stopped having sex since the prior interview (reported sex in the prior interview, but reported no sex in the current interview) and 7.62% (4389/57,602) reported that they started having sex (reported no sex in the prior interview, but reported sex in the current interview). In terms of partner transitions, 4.08% (2353/57,602) of interviews indicated a break up (partner reported in the prior interview, but no partner reported in current interview), 4.08% (2353/57,602) indicated a new partner (no partner in the prior interview, partner in the current interview), and 2.69% (1548/57,602) indicated a partner switch (partner in the prior interview, different partner in the current interview). In terms of contraceptive use, 4.51% (2595/57,602) of interviews indicated stopping (contraceptive use in prior interview, no use in current interview) and 4.33% (2496/57,602) indicated starting (no contraceptive use in prior interview, use in current interview). Finally, 0.49% (284/57,602) of interviews indicated the end of a pregnancy (pregnant in prior interview, not pregnant in current interview) and 0.44% (254/57,602) indicated a new pregnancy (not pregnant in prior interview, pregnant in current interview).

We included these time-varying measures in our models that used the 57,602 journal interviews as the unit of observation (presented in Multimedia Appendix 4). Since each respondent will have experienced multiple changes between multiple pairs of journal interviews, we cannot include these measures of change in models using respondents as the unit of analysis (presented in Multimedia Appendix 3), but include instead the three summary measures of change from the bottom of Multimedia Appendix 2.

#### Dependent Variables and Analytic Methods

We used three dependent variables in our analyses: total days in the study for each respondent, total number of journal interviews completed by each respondent, and whether each journal interview was completed late.

**Figure 3:**
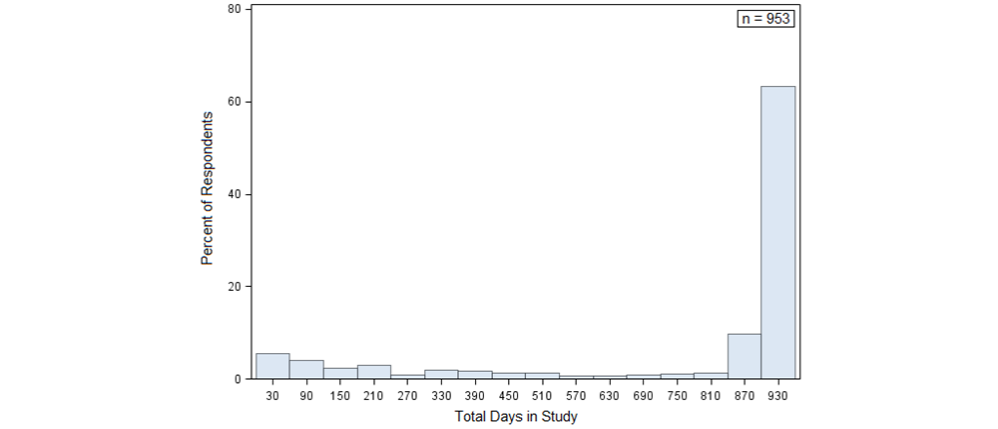
Distribution of total days in study.

Total days in study is a measure of attrition, and indicates the elapsed time from the first interview to the last interview. The mean days in study is 740.70, slightly more than two years, and ranges from 8 to 954 days. Figure 3 illustrates the distribution of this variable. Attrition is slightly higher in the first month, but declines steadily until 840 days (2.30 years). More than 63.4% (604/953) of the young women completed their final interview at 900 or more days (2.47 years). We used ordinary least squares (OLS) regression to model this dependent variable, with results presented in Multimedia Appendix 3.

**Figure 4:**
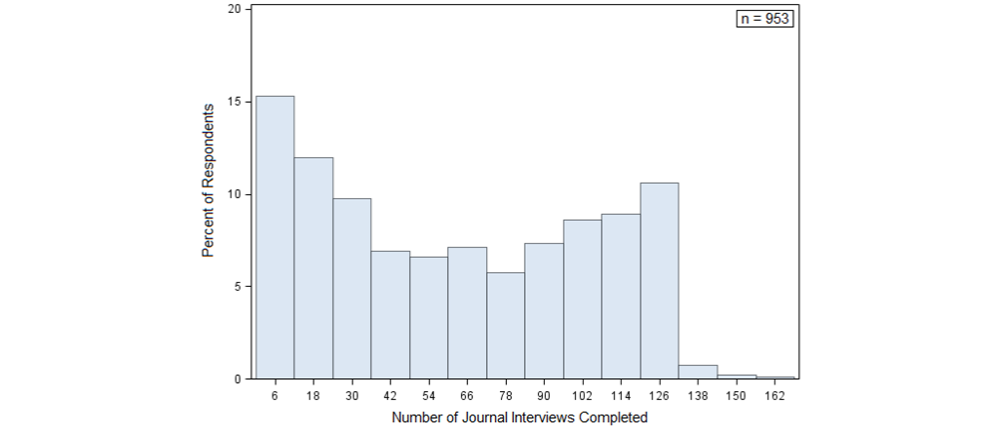
Distribution of total number of journals completed.

The total number of journal interviews completed is a count of how many interviews the respondent completed. Among those who completed at least one journal interview after enrollment, the mean number of journals completed was 61.45, and ranges from 2 to 165. Approximately 15% of respondents completed fewer than 12 journal interviews. The target number of journals, which would require one every seven days, was 130. Figure 4 shows the distribution for this variable. As mentioned above, we encouraged respondents to complete journals every 7 days, but journals could be completed as early as 5 days after the previous journal was completed. Some respondents did choose to complete their journals more frequently than weekly and so they ended the study with more than 130 journals. We used OLS regression to estimate models of this dependent variable in Multimedia Appendix 3.

Finally, we looked at late completions, defining a *late* interview as one that occurred 14 or more days after the prior interview. An interview completed within 13 days still refers to changes since the prior interview, where at >14 days respondents were instructed to adjust the reference period to solely the week before, causing a period of missing data. As indicated in Table 2, only 11.07% (6376/57,602) of journals were completed late. We used logistic regression to estimate models of this dependent variable in Multimedia Appendix 4. This measure is specific to the journal interview, and it allows for an analysis of whether events that occurred just prior to the interview may have influenced the probability of late completion.

**Table 2:** Descriptive statistics for dependent variables

	Mean/ Proportion	SD	Range
**Dependent Variables**			
Total days in study (n=953)	740.70	303.68	8 – 954
Total number of journals completed (n=953)	61.45	42.54	2 – 165
Journal completed late (n=57,602)	.11	---	0 – 1

We used multilevel logistic regression (estimated using the SURVEYLOGISTIC procedure in Statistical Analysis System) to estimate the log-odds of the interview being late, which accounted for the multiple journal interviews for each respondent. This model takes the form 

 logit(p_it_)=β_0t_ + γ*X_i_ + δ*Z_it_

where p_it_ is the probability of a journal interview being late, **X**_i_ is a set of non-time-varying characteristics of individual i, and **Z**_it_ is a set of time-varying characteristics associated with journal interview t, for each individual i. Thus, the model estimated effects on the probability of a particular journal interview being late as a function of both the characteristics of the individual completing the interview (**X**_i_) and the events that occurred since the prior journal interview (**Z**_it_).

## Results

Multimedia Appendix 3 presents results for models predicting total days in the study (attrition) and total number of journal interviews completed. Results in columns 1 and 3 are for models using baseline measures only, while columns 2 and 4 are for models that add the three summary measures of experiences over the entire study period (number of new sexual partners, had sex without contraception, any pregnancy). These nested models allowed us to determine whether the relationship between baseline characteristics and participation were net of experiences during the study.

### Total Days in Study

In the models of attrition shown in columns 1 and 2 of Multimedia Appendix 3, positive numbers indicate more days in the study and negative numbers indicate fewer days in the study. Column 1 shows that African American women remained in the study for 45 fewer days than white women. Young women with less education, and/or a mother who was younger than 20 at her first birth, remained in the study for fewer days than their more advantaged peers. Highly religious respondents remained in the study longer than their less religious peers. Personality characteristics did not predict attrition. Respondents who provided both an email address and a phone number stayed in the study nearly four months (114 days) longer than those who provided only one or no type of contact information. Entering the study with a prior pregnancy was not associated with attrition, but respondents who had two or more prior pregnancies remained in the study for approximately 90 fewer days than those with no prior pregnancies.

The model shown in column 2 of Multimedia Appendix 3, which includes the three summary measures of change, indicates that respondents who experienced changes during the study period remained in the study for a substantially longer period than their peers who experienced fewer or no changes. Respondents with more new sexual partners during the study period stayed in the study longer; on average, each new sex partner resulted in 27 additional days. Those who had sex without contraception stayed in the study for an average of 87 days longer than their peers who did not have sex without contraception. Additionally, respondents who reported a pregnancy remained in the study for 141 days longer than those who did not report a pregnancy. The effects of sociodemographic background, personality characteristics, pregnancy-related experiences that occurred prior to the study, and contact information on the total number of days in the study are largely *net of* experiences during the study. The effects of experiences during the study are *net of* sociodemographics, personality, contact information, and prior experiences.

Note that because the dependent variable is not normally distributed, we also used logistic regression to model the probability of remaining in the study for at least 741 days (the mean value of the continuous variable). Results did not differ, so we present the OLS models due to their simpler interpretation.

### Total Number of Completed Journals

Columns 3 and 4 in Multimedia Appendix 3 present OLS regression models of the total number of journal interviews completed by each respondent. This dependent variable represents both continued participation (total length of time in study is related to number of interviews completed) and timeliness (length of intervals between interviews is related to number of interviews completed). As such, it captures the quantity and quality of participation and, ultimately, the volume of information provided by respondents. In these models, positive coefficients indicate a larger number of completed journals at the end of the respondent’s participation in the study, and negative coefficients indicate a smaller number of journals.

It is not surprising that some of the respondent characteristics that predict attrition were also associated with total number of journals completed, given the relationship between these two variables. In addition to predicting fewer total days in the study, the following characteristics also predicted completing fewer journals: being African American, completing high school but not currently enrolled in school, having a mother whose first birth was before age 20, not providing detailed contact information (both phone and email), and having risky adolescent experiences with sex and pregnancy (sex without contraception, and/or two or more pregnancies). However, column 3 shows that the following factors that predict total days in the study *were not* associated with total interviews completed: being enrolled in high school (relative to enrolled in a 4-year college), being highly religious, and growing up with a single parent. Conversely, having dropped out of high school (relative to enrolled in a 4-year college), growing up with an *other* parental living arrangement (relative to two parents), and extraversion all predicted completing fewer journal interviews, but had no association with total days in the study. Finally, conscientiousness was associated with completing almost 6 more interviews, but did not predict days in the study.

Column 4 of Multimedia Appendix 3 shows that changes in sex and pregnancy experienced during the study were strong predictors of the total number of completed journals. Respondents with more new sexual partners and pregnancy (or pregnancies) during the study period completed more journal interviews; on average, each new sex partner resulted in nearly 4 additional journals, and those who experienced a pregnancy completed almost 7 more journals than those who did not. As was the case for models of total days in the study, the effects of sociodemographics, personality, contact information, and prior experiences on the total number of journal interviews were largely *net of* experiences during the study, and the effects of experiences during the study were net of sociodemographics, personality, contact information, and prior experiences.

### Late Journal Completion

Multimedia Appendix 4 presents models of whether each journal interview was completed late (>14 days since the prior interview). The unit of analysis is the journal interview rather than the respondent, therefore these models allowed us to examine if interviews were more likely to be late when changes in pregnancy-related behaviors were reported.

The coefficients in Multimedia Appendix 4 are the multiplicative effects on the odds of late journal completion (odds ratios). Thus, a coefficient of 1.00 indicates no effect, a coefficient less than 1.00 indicates lower odds of a late journal, and a coefficient greater than 1.00 indicates higher odds of a late journal. Column 1 presents results from the model based on respondent sociodemographic and personality characteristics, contact information/mode, and adolescent (pre-study) pregnancy-related experiences. The model shown in column 2 adds pregnancy-related changes since the prior journal.

A few background factors were associated with late journal completion: journals completed by African-American and/or less educated respondents were more frequently late than those completed by white/more-educated respondents. Journals completed by more conscientious respondents were less frequently late than those completed by less conscientious respondents. Respondents who provided both an email address and a phone number were less frequently late than those who provided only one or no type of contact information. Those who had sex without contraception during adolescence were more frequently late.

Pregnancy-related changes since the prior journal interview were strong and significant predictors of whether the subsequent journal interview was completed late. Weeks in which the respondent stopped having sex, started having sex, broke up with a partner, got a new partner, switched partners, stopped using contraception, started using contraception, got pregnant, or ended a pregnancy were all associated with late completion of the interview reporting these changes. Recall, however, that having these experiences throughout the study period was associated with less attrition from the study. Thus, although respondents with these experiences remained in the study longer, and completed more journal interviews, the interviews in which these behaviors were reported were more likely to be completed late.

## Discussion

In sum, our findings on duration of participation in an intensive longitudinal study are consistent with more general findings on survey participation: minority and lower socioeconomic status respondents remained in the study for a shorter period than others [11,23,24], and also completed fewer journals and had higher odds of late journal completion. Personality factors (extraversion, agreeableness, conscientiousness, neuroticism, and intellect/imagination) were not associated with overall time in the study, but extraverted and less conscientious respondents completed fewer interviews. Conscientious respondents also had lower odds of late journal completion. We found that contact information provided by the respondent was an important factor, with respondents who provided both an email address and a phone number remaining in the study longer, completed more journal interviews, and completed those interviews on time, compared to those who provided only one (or no) contact mode. This finding is consistent with existing research suggesting that pre-existing (at the time the study began) panel study commitment is a strong predictor of attrition [11]. We suspect that respondents who were more committed to participating in the RDSL study provided both email and phone contact information, while those less committed provided only one method, and those least committed provided no contact information.

Of greatest interest to our study, we found that experiences related to pregnancy were strong predictors of attrition and timeliness. Sex without contraception and two or more pregnancies before the study (during adolescence) were associated with higher levels of attrition and fewer total journal interviews during the study. Although these risky behaviors before the study were associated with attrition and fewer interviews, respondents with many changes in these behaviors during the study actually remained in the study longer and completed more interviews than their counterparts. This finding was reassuring since the RDSL was designed to investigate factors shaping the dynamics of sexual behavior, contraceptive use, and pregnancy. The specific weeks when changes in those risky sexual behaviors were reported, however, were more likely to be late (14 or more days since the prior interview) compared to weeks that continued the prior week’s behavior. This trend may be consistent with other research finding that *shocks* (typically life events or negative experiences with the survey) can lead to attrition [33]. In this study, although these experiences did not seem to increase attrition, they did delay survey responses. These results may also be consistent with research finding that health status predicts attrition [24], although that research did not specifically address pregnancy as a health status.

Our finding that respondents with dynamic experiences that were measured in the surveys remained in the study longer and completed more journal interviews is consistent with studies finding that boring survey modules increase attrition [11], and that tailored questionnaire content reduces attrition [21]. Further, we know from semi-structured interviews and an open-ended question at the end of each weekly journal interview that some respondents found the long-term and repetitive nature of the journal interviews tedious. This problem is highlighted by several responses to the weekly question, “Is there anything else you would like to tell us?” Responses included, “I think that you guys need to ask different questions. I am answered (sic) the same questions over and over again.”, “No but I think you should make the survey shorter or combine it to a couple of pages.”, and “Same questions every week!!! Come on.”

We speculate, therefore, that perhaps the frequent interviews may have seemed less burdensome for respondents whose lives included more changes in the experiences that were the focus of the study. Changes in these experiences would result in more varied survey content from week to week. This issue was also hinted at in some of the responses to the open-ended question at the conclusion of the journal, for example, “Doing this survey helps me to vent quite a bit because I don’t have many people around me that I can tell the whole truth to. So thank you for this opportunity…”

Overall, our analyses suggest that attrition was a relatively smooth process in our study, with slightly higher rates in the first month, but then low rates that increased slowly and steadily until the end of the study. Further, the vast majority of respondents (90.7%, 865/953) completed at least half of their journal interviews on time (before 14 days had passed).

Our study focused on a young age group (18 and 19 years old at the beginning of the study, and 20 to 22 years at the end) that typically has higher attrition than older adults [23], suggesting that our attrition rate may have been higher than similar studies of older age groups would be. However, given that tailored content can reduce attrition, and our substantive topics were so important to this age group, our study may have had lower attrition than other studies. We present this as a case study, with the hope that further studies will explore attrition in surveys about other topics, and with other age groups.

### Limitations and Future Research

This study of continued and on-time participation in a weekly survey, as well as the RDSL study more generally, has several important limitations. Most significant, we lack crucial information about respondents who dropped out of the study. Although we can summarize changes experienced during the period that attriters remained in the study, we do not know what events occurred just prior to their decisions to stop participating. For example, although we found that respondents dropped out at higher rates after reporting a pregnancy, some respondents may have left the study when they discovered they were pregnant but had not yet reported it. We do not believe that this is the case, considering our study’s pregnancy rates closely resemble the vital statistics rates for this age group in this area, but we cannot rule out this possibility. Further, we do not know what other unmeasured experiences may have contributed to respondents’ decisions to drop out.

The narrow geographic focus (a single county in Michigan) of the RDSL study is also a limitation. However, although the sample is not representative of the United States as a whole, Michigan falls close to the national median for many of the measures of interest in this study: cohabitation, marriage, age at first birth, completed family size, non-marital childbearing, and teenage childbearing [34]. This information does not suggest that Michigan is representative of the nation, but implies that it is not an outlier. Another constraint is that the study includes only a small number of Latinas, which is a growing demographic group in the United States. We hope that the research findings of the RDSL will motivate future researchers to implement journal methods on larger and more diverse populations.

This study focused on a narrow age range, and a set of topics that were of great importance to the study population. It is difficult to know how these general conclusions about attrition – that those with the most experiences related to the subject of the study participate longer and complete more interviews, but have higher odds of late interviews – would translate to surveys on other age groups or on other topics. It is possible that studies focused on topics of specific interest to the age group in the study might have less attrition than others.

Finally, our study design did not include experiments related to reducing attrition. With a few minor differences, all respondents were subject to the reminder protocol described in Figure 2, and the incentive protocol described above, across all weeks in the study. Additionally, all respondents were asked to complete the surveys weekly and so we do not know if participation would have been different if we had asked some or all respondents to complete the surveys less frequently. Future intensive longitudinal surveys should include experiments designed to test and optimize reminder and incentive protocols, and to determine the ideal frequency of measurement.

Experiments are also needed to determine how to keep respondents engaged in intensive longitudinal surveys. In this study, the complicated nature of the association between pregnancy-related behaviors and study participation – the reduced timeliness of interviews during weeks of change, but the larger number of interviews for respondents who experienced more changes in these behaviors – highlights the need for responsive designs in social research. For example, respondents who are not sexually active might be more engaged if the frequency of interviews was reduced to monthly, or if interview content could be tailored to better reflect their experiences. Respondents who are sexually active, and who may have trouble completing their journal interviews during particularly busy weeks of their lives, may find it easier to participate via a mobile version of the journal interview. In fact, if we were to conduct this study again today, we would have a mobile site or app available to complete the survey, given current knowledge and the increase in smartphone and mobile device use among this population [35].

### Conclusions

We have demonstrated in this paper that participation in an intensive longitudinal study with weekly web surveys may vary not only by respondent characteristics, but also by behaviors measured across the surveys. Our analyses suggest that respondents who experience the behaviors measured by the study may participate more than respondents who do not experience those behaviors. These results highlight the need for experiments to determine the most effective data collection strategies and procedures for assuring continued and on-time participation in intensive longitudinal studies.

## Appendix

Multimedia Appendix 1 Sample journal interviews.

Multimedia Appendix 2 Descriptive statistics for individual characteristics (N=953).

Multimedia Appendix 3 OLS regression models of total days in study and total number of journals completed (n=953 respondents).

Multimedia Appendix 4 Logistic regression models of late journal completion (coefficients are odds ratios; confidence intervals in parentheses; n=953 respondents; n=57,602 journal interviews).

## Abbreviations

NEO-FFI: Neuroticism, Extraversion, and Openness to Experience-Five Factor Inventory
OLS: Ordinary least squares
OR: odds ratio
PID: Personal Identification Card
RDSL: Relationship Dynamics and Social Life
