# Supplementary material for: Participation in an Intensive Longitudinal Study with Weekly Web Surveys Over 2.5 Years
Source: J Med Internet Res. 2016 Jun 23;18(6):e105. doi: 10.2196/jmir.5422 (PMC4937177; doi:10.2196/jmir.5422)
Supplement: Multimedia Appendix 1 [file jmir_v18i6e105_app1.pdf]

Sample Journal Interview #1

(Sample questions and responses for a respondent not in a relationship)

For these first questions, please give a number from 0 to 100, where 0 means that you think there is absolutely no chance, and 100 means that you think the event is absolutely sure to happen. And you can give any number from 0 to 100.

If you were to have sexual intercourse <u>regularly</u>, say once a week for a year, <u>without using birth control</u>, what do you think are the chances that you would get pregnant?

[90] 0-100 chance

If you were to have unprotected sexual intercourse with someone you just met, what do you think are the chances that you would get HIV or AIDS?

[60] 0-100 chance

What are the chances that you will have a baby while not married?

[10] 0-100 chance

What are the chances that you will get HIV or AIDS in your lifetime?

[15] 0-100 chance

What are the chances that you will graduate from college?

[95] 0-100 chance

What are the chances that you will have a middle-class family income by age 30?

[75] 0-100 chance

If you were to have sexual intercourse <u>once or twice without using birth control</u>, what are the chances that you would get pregnant?

[65] 0-100 chance

Do you think there might be a chance that you are pregnant right now?

- ○ Yes
- ◉ No

How much do you want to get pregnant during the next month? Please give a number between 0 and 5, where 0 means you don’t at all want to get pregnant and 5 means you really want to get pregnant.

0

How much do you want to avoid getting pregnant during the next month? Please give a number between 0 and 5, where 0 means you don’t at all want to avoid getting pregnant and 5 means you really want to avoid getting pregnant.

5

Since your last interview on October 20, did you use or do anything that can help people avoid becoming pregnant, even if you did not use it to keep from getting pregnant yourself?

- ○ Yes
- ◉ No

In the past 7 days (since October 20), have you had a special romantic relationship with anyone?

- ○ Yes
- ◉ No

In the past 7 days (since October 20), have you had physical or emotional contact, such as kissing, dating, spending time together, sex, or other activities with a partner?

- ○ Yes
- ◉ No

Is there anything else that has happened since your last interview on October 20 that you would like to report?

No.

Thank you for your participation in this week’s survey. You have earned $10 so far.

The invitation to complete you next weekly survey will be sent to you in 7 days.

Please click on the “Submit” button below to complete the survey. Once your answers are submitted, they will be locked to maintain your privacy.

Sample Journal Interview #2

(Sample questions and responses for a pregnant respondent not in a relationship)

Last interview you reported that you were pregnant. Are you still pregnant?

- (•) Yes
- ( ) No

Now, think about the past seven days. How often was each of the following things true during the past seven days?

You felt that you could not shake off the blues, even with help from your family and your friends. Was this never true, almost never true, sometimes true, fairly often true, or very often true?

- (•) Never True
- ( ) Almost never true
- ( ) Sometimes true
- ( ) Fairly often true
- ( ) Very often true

You felt depressed. Was this never true, almost never true, sometimes true, fairly often true, or very often true?

- ( ) Never true
- ( ) Almost never true
- ( ) Sometimes true
- ( ) Fairly often true
- (•) Very often true

You felt sad. Was this never true, almost never true, sometimes true, fairly often true, or very often true?

- ( ) Never true
- ( ) Almost never true
- (•) Sometimes true
- ( ) Fairly often true
- ( ) Very often true

You felt life was not worth living. Was this never true, almost never true, sometimes true, fairly often true, or very often true?

- (•) Never true
- ( ) Almost never true
- ( ) Sometimes true
- ( ) Fairly often true
- ( ) Very often true

You were happy. Was this never true, almost never true, sometimes true, fairly often true, or very often true?

- ( ) Never true
- ( ) Almost never true
- (•) Sometimes true
- ( ) Fairly often true
- ( ) Very often true

In the past 7 days (since October 20), have you had a special romantic relationship with anyone?

- ( ) Yes
- (•) No

In the past 7 days (since October 20), have you had physical or emotional contact, such as kissing, dating, spending time together, sex, or other activities with a partner?

- ( ) Yes
- (•) No

In the past 7 days (since October 20), have you had any contact with AA?

- ( ) Yes
- (•) No

In the past 7 days (since October 20), who of the following have you talked to about your pregnancy? [CHECK ALL THAT APPLY]

- (•) Mother
- (•) Father
- ( ) Step-mother
- ( ) Step-father
- (•) Brother
- ( ) Sister
- (•) Grandparent
- ( ) Aunt
- ( ) Uncle
- ( ) Cousin
- (•) Friend
- ( ) No one
- ( ) Other [SPECIFY] ________

In the past 7 days (since October 20), who of the following gave you money or bought things for you? [CHECK ALL THAT APPLY]

- ( ) Mother
- (•) Father
- (•) Step-mother
- ( ) Step-father
- ( ) Brother
- ( ) Sister
- (•) Grandparent
- (•) Aunt
- ( ) Uncle
- ( ) Cousin
- ( ) Friend
- ( ) No one
- ( ) Other [SPECIFY] ________

In the past 7 days (since October 20), who of the following helped you in other ways, such as providing transportation to a pre-natal clinic or helping with errands? [CHECK ALL THAT APPLY]

- (•) Mother
- ( ) Father
- ( ) Step-mother
- ( ) Step-father
- ( ) Brother
- ( ) Sister
- ( ) Grandparent
- ( ) Aunt
- ( ) Uncle
- ( ) Cousin
- (•) Friend
- ( ) No one
- ( ) Other [SPECIFY] ______

In the past 7 days (since October 20), did you see a doctor, nurse, or other medical professional about your pregnancy?

- ( ) Yes
- (•) No

In the past 7 days (since October 20), did you have any problems or complications with your pregnancy?

- ( ) Yes
- (•) No

In the past 7 days (since October 20), did you smoke cigarettes?

- ( ) Yes
- (•) No

In the past 7 days (since October 20), did you drink alcohol?

- (•) Yes
- ( ) No

In the past 7 days (since October 20), did you use marijuana?

- ( ) Yes
- (•) No

In the past 7 days (since October 20), did you use any other types of illegal drugs, such as LSD, PCP, ecstasy, mushrooms, inhalants, crystal meth, ice, cocaine, crack, heroine, or prescription medicines not prescribed to you?

- ( ) Yes
- (•) No

In the past 7 days (since October 20), how often have you felt that you were unable to control the important things in your life? Would you say never, almost never, sometimes, fairly often, or very often?

- ( ) Never
- ( ) Almost never
- (•) Sometimes
- ( ) Fairly often
- ( ) Very often

In the past 7 days (since October 20), how often have you felt confident in your ability to handle your personal problems?

- ( ) Never
- ( ) Almost never
- (•) Sometimes
- ( ) Fairly often
- ( ) Very often

In the past 7 days (since October 20), how often have you felt that things were going your way?

- ○ Never
- ○ Almost never
- ◉ Sometimes
- ○ Fairly often
- ○ Very often

In the past 7 days (since October 20), how often have you felt difficulties were piling up so high that you could not overcome them?

- ○ Never
- ◉ Almost never
- ○ Sometimes
- ○ Fairly often
- ○ Very often

Is there anything else that has happened since your last interview on October 20 that you would like to report?

No.

Thank you for participating in this week's survey. You have earned $10 so far.

The invitation to complete your next weekly survey will be sent to you in 7 days.

Please click on the "Submit" button below to complete the survey. Once your answers are submitted, they will be locked to maintain your privacy.

Sample Journal Interview #3
(Sample questions and responses for a respondent in a relationship)

First, a few questions about your friends.

How many of your friends have had sexual intercourse? Would you say none, a few, many, or almost all of them?

- ○ None
- ○ A few
- ◉ Some
- ○ Many
- ○ Almost all

How many of your friends are using birth control?

- ○ None
- ○ A few
- ○ Some
- ◉ Many
- ○ Almost all

How many of your friends have had sexual intercourse without using birth control?

- ○ None
- ◉ A few
- ○ Some
- ○ Many
- ○ Almost all

How many of your friends have gotten pregnant?

- ◉ None
- ○ A few
- ○ Some
- ○ Many
- ○ Almost all

How many of your fiends are attending or planning to attend college?

- ○ None
- ○ A few
- ○ Some
- ◉ Many
- ○ Almost all

How many of your friends are parents?

- ◉ None
- ○ A few
- ○ Some
- ○ Many
- ○ Almost all

How many of your friends are working full time?

- ○ None
- ◉ A few
- ○ Some
- ○ Many
- ○ Almost all

How many of your friends dropped out of high school?

- ◉ None
- ○ A few
- ○ Some
- ○ Many
- ○ Almost all

Next is a question about your community. How many women in your community are single parents?

- ◯ None
- ◉ A few
- ◯ Some
- ◯ Many
- ◯ Almost all

Do you think there might be a chance you are pregnant now?

- ◯ Yes
- ◉ No

How much do you want to get pregnant during the next month? Please give a number between 0 and 5, where 0 means you don't at all want to get pregnant and 5 means you really want to get pregnant.

0

How much do you want to avoid getting pregnant during the next month? Please give a number between 0 and 5, where 0 means you don't at all want to avoid getting pregnant and 5 means you really want to avoid getting pregnant.

5

Since your last interview on October 20, did you use or do anything that can help people avoid becoming pregnant, even if you did not use it to keep from getting pregnant yourself?

- ◉ Yes
- ◯ No

At your last interview, you indicated that you were in a special romantic relationship with AB. Are you still in a relationship with AB?

- ◉ Yes
- ◯ No

In the past 7 days (since October 20), did you and AB spend a lot of time together?

- ◉ Yes
- ◯ No

Have you and AB agreed to only have a special romantic relationship with each other, and no one else?

- (•) Yes
- ( ) No

In the past 7 days (since October 20), have you talked with AB about birth control?

- ( ) Yes
- (•) No

How much do you think AB wants you <u>to</u> get pregnant during the next month? Please give a number between 0 and 5, where 0 means AB doesn't want you to get pregnant and 5 means AB really wants you to get pregnant.

0

How much do you think AB wants you to <u>avoid</u> getting pregnant during the next month? Please give a number between 0 and 5, where 0 means AB doesn't at all want you to avoid getting pregnant and 5 means AB really wants you to avoid getting pregnant.

5

In the past 7 days (since October 20), has AB told you that during the upcoming month AB wants you to get pregnant?

- ( ) Yes
- (•) No

Who decides what to do or where to go when you go out? Would you say you do, AB does, or do you both decide?

- ( ) You
- ( ) AB
- (•) Both decide

In the past 7 days (since October 20), did you and AB fight or have any arguments?

- ( ) Yes
- (•) No

Since your last interview on October 20, did you get married to AB?

- ( ) Yes
- (•) No

Since your last interview on October 20, did you get engaged to AB?

- ○ Yes
- ● No

In the past 7 days (since October 20), how many nights did you spend all night sleeping in the same bed with AB?

1

Do you have a place you live that is separate from where AB lives?

- ● Yes
- ○ No

In the past 7 days (since October 20), did you have sexual intercourse with AB? By sexual intercourse, we mean when a man puts his penis into a woman's vagina.

- ● Yes
- ○ No

In the past 7 days (since October 20), did you have sexual intercourse with anyone other than AB?

- ○ Yes
- ● No

In the past 7 days (since October 20), do you think AB has had sexual intercourse with anyone other than you?

- ○ Yes
- ● No

How much do you want to get pregnant with AB during the next month? Please give a number between 0 and 5, where 0 means you don't at all want to get pregnant with AB and 5 means you really want to get pregnant with AB.

1

How much do you want to avoid getting pregnant with AB during the next month? Please give a number between 0 and 5, where 0 means you don't at all want to avoid getting pregnant with AB and 5 means you really want to avoid getting pregnant with AB.

4

If you were to get pregnant in the upcoming month, would you move in with AB?

- (•) Yes
- ( ) No

If you were to get pregnant in the upcoming month, would you get married to AB?

- ( ) Yes
- (•) No

Is there anything else that has happened since your last interview on October 20 that you would like to report?

No.

Thank you for participating in this week's survey. You have earned $10 so far.

The invitation to complete your next weekly survey will be sent to you in 7 days.

Please click on the "Submit" button below to complete the survey. Once your answers are submitted, they will be locked to maintain you privacy.
