# Supplementary material for: Participation in an Intensive Longitudinal Study with Weekly Web Surveys Over 2.5 Years
Source: J Med Internet Res. 2016 Jun 23;18(6):e105. doi: 10.2196/jmir.5422 (PMC4937177; doi:10.2196/jmir.5422)
Supplement: Multimedia Appendix 2 [file jmir_v18i6e105_app2.pdf]

Multimedia Appendix 2. Descriptive Statistics for Individual Characteristics (N=953)

	Mean/ Proportion	*SD*
**Sociodemographic Characteristics**		
African American	.34	
Education		
Enrolled in high school	.14	
Enrolled in 2-year college or vocational program	.29	
Enrolled in 4-year college	.28	
Completed high school/not enrolled	.22	
Dropped out of high school/not enrolled	.08	
Receiving public assistance	.26	
Religious importance (1=not important, 4=more important than anything else)	2.69	
Biological mother less than 20 years old at first birth	.37	
Family Structure		
Two parents	.52	
One biological parent only	.40	
Other	.08	
Mother’s education less than high school graduate	.08	
Parent’s income		
<$15,000	.14	
$15,000 to $44,999	.28	
$45,000 to $74,999	.19	
$75,000 or greater	.19	
Don’t know/Refused	.20	
Age	19.19	.57
**Personality**		
Extraversion	2.69	.53
Agreeableness	3.07	.42
Conscientiousness	2.72	.47
Neuroticism	2.49	.46
Intellect/Imagination	2.91	.42
**Contact Information/Mode**		
Contact information: provided email and phone	.84	
Reminder mode: text and email	.33	
Journal interview completed by phone (n=57,602)	.12	
**Adolescent Experiences Related to Pregnancy**		
Age at first sex 16 years or less	.52	
Number of sexual partners 2 or more	.60	
Ever had sex without contraception	.48	
Number of prior pregnancies		
Zero	.74	
One	.17	
Two or more	.09	
**Summary of Changes During Study Period**		
Number of (new) sexual partners	1.83	1.91
Any sex without contraception	.54	
Any pregnancy	.21	
