# Supplementary material for: Participation in an Intensive Longitudinal Study with Weekly Web Surveys Over 2.5 Years
Source: J Med Internet Res. 2016 Jun 23;18(6):e105. doi: 10.2196/jmir.5422 (PMC4937177; doi:10.2196/jmir.5422)
Supplement: Multimedia Appendix 3 [file jmir_v18i6e105_app3.pdf]

Multimedia Appendix 3. OLS Regression Models of Total Days in Study and Total Number of Journals Completed (n=953 respondents)

	Total Days in Study				Total Number of Completed Journals			
	1	*P*	2	*P*	3	*P*	4	*P*
**Sociodemographic characteristics**								
African American (ref: white)	-45.37 (24.88)	.07	-48.38 (23.34)	.04	-10.92 (3.25)	<.001	-11.35 (3.16)	<.001
Education (ref: enrolled in 4-year college)								
Enrolled in high school	-67.63 (34.44)	.049	-80.53 (32.42)	.01	-6.15 (4.49)	.17	-6.99 (4.38)	.11
Enrolled in 2-year college or vocational program	-14.26 (26.32)	.59	-22.12 (24.76)	.37	-3.15 (3.43)	.36	-3.88 (3.36)	.25
Completed high school/not enrolled	-51.39 (29.89)	.09	-71.01 (28.18)	.01	-11.47 (3.90)	.003	-12.29 (3.82)	.001
Dropped out of high school/not enrolled	-39.46 (42.98)	.36	-34.64 (40.42)	.39	-15.32 (5.61)	.006	-14.90 (5.48)	.007
Receiving public assistance	-4.92 (27.31)	.86	2.43 (25.67)	.92	-1.00 (3.56)	.78	0.03 (3.48)	.99
Religious importance	23.30 (11.58)	.04	26.22 (10.90)	.02	-.05 (1.51)	.97	.54 (1.48)	.72
Biological mother less than 20 years old at first birth	-50.68 (21.51)	.02	-47.62 (20.20)	.02	-7.13 (2.81)	.01	-6.52 (2.74)	.02
Family Structure (ref: two parents)								
One biological parent only	-37.08 (22.57)	.10	-41.70 (21.17)	.049	-4.21 (2.95)	.15	-4.49 (2.87)	.12
Other	-58.49 (38.71)	.13	-57.93 (36.33)	.11	-8.89 (5.05)	.08	-8.47 (4.92)	.09
Mother's education less than high school	-25.89 (35.78)	.47	-32.25 (33.58)	.34	-7.23 (4.67)	.12	-7.37 (4.55)	.11
Parent's income (ref: < $15,000)								
$15,000 to $44,999	24.64 (32.53)	.45	-23.62 (30.54)	.44	3.50 (4.25)	.41	2.97 (4.14)	.47
$45,000 to $74,999	1.21 (36.53)	.97	-3.92 (34.33)	.91	-2.43 (4.77)	.61	-3.63 (4.65)	.44
$75,000 or greater	-18.72 (39.03)	.63	-8.33 (36.69)	.82	1.60 (5.09)	.75	1.73 (4.97)	.73
Don't know/Refused	37.55 (33.69)	.27	31.99 (31.64)	.31	6.62 (4.40)	.13	5.56 (4.29)	.20
Age	-26.73 (18.04)	.14	-14.09 (16.96)	.41	-.96 (2.35)	.68	.22 (2.30)	.92
**Personality**								
Extraversion	-9.50 (19.50)	.63	-26.28 (18.35)	.15	-6.40 (2.54)	.01	-7.88 (2.49)	.002
Agreeableness	15.18 (25.91)	.56	2.14 (24.34)	.93	3.12 (3.38)	.36	1.85 (3.30)	.57
Conscientiousness	9.09 (21.16)	.67	13.48 (19.87)	.049	5.82 (2.76)	.04	6.48 (2.69)	.02
Neuroticism	-8.70 (22.64)	.70	-14.05 (21.37)	.51	-1.31 (2.95)	.66	-1.74 (2.88)	.55
Intellect/Imagination	7.74 (24.19)	.75	19.04 (22.72)	.40	.11 (3.16)	.97	.75 (3.08)	.81

**Contact Information/Mode**								
Contact information: provided email and phone (ref: provided only one or no contact information)	114.03 (28.69)	<.001	93.97 (26.97)	<.001	17.99 (3.74)	<.001	16.26 (3.66)	<.001
Reminder mode: text and email (ref: only one or no mode for reminder)	-13.19 (20.89)	.53	-23.98 (19.62)	.22	-.88 (2.73)	.75	-1.96 (2.66)	.46
**Adolescent Pregnancy-Related Experiences**								
Age at first sex 16 years or less	-16.46 (25.94)	.53	-30.95 (24.39)	.20	-2.84 (3.38)	.40	-3.85 (3.31)	.24
Number of sexual partners 2 or more	-1.70 (26.67)	.95	-46.43 (25.56)	.07	-5.01 (3.48)	.15	-9.65 (3.46)	.005
Ever had sex without contraception	-34.59 (24.22)	.15	-64.41 (22.89)	.005	-7.74 (3.16)	.01	-9.92 (3.10)	.001
Number of pregnancies (ref: zero)								
One	-1.28 (30.71)	.97	-26.02 (28.97)	.37	-2.91 (4.01)	.47	-4.06 (3.93)	.30
Two or more	-89.62 (38.53)	.02	-111.11 (36.24)	.002	-15.74 (5.03)	.002	-16.75 (4.91)	<.001
**Summary of Changes During Study Period**								
Number of (new) sexual partners			26.71 (5.20)	<.001			3.82 (.70)	<.001
Any sex without contraception			87.26 (21.06)	<.001			4.70 (2.85)	.10
Any pregnancy			140.79 (24.30)	<.001			6.64 (3.29)	.04
R^2 (adjusted)	.08		.19		.20		.24	
