# Supplementary material for: Participation in an Intensive Longitudinal Study with Weekly Web Surveys Over 2.5 Years
Source: J Med Internet Res. 2016 Jun 23;18(6):e105. doi: 10.2196/jmir.5422 (PMC4937177; doi:10.2196/jmir.5422)
Supplement: Multimedia Appendix 4 [file jmir_v18i6e105_app4.pdf]

Multimedia Appendix 4. Logistic Regression Models of Late Journal Completion (coefficients are odds ratios, confidence intervals in parentheses) (n=953 respondents, n=57,602 journal interviews)

	Late Journal Completion			
	1	*P*	2	*P*
**Sociodemographic Characteristics**				
African American	1.34 (1.10 – 1.62)	.004	1.31 (1.08 – 1.59)	.007
Education (ref: enrolled in 4-year college)				
Enrolled in high school	1.02 (.77 – 1.36)	.89	1.00 (.75 – 1.33)	.98
Enrolled in 2-year college or vocational program	1.07 (.86 – 1.34)	.55	1.06 (.85 – 1.33)	.59
Completed high school/not enrolled	1.30 (1.01 – 1.67)	.04	1.30 (1.01 – 1.66)	.04
Dropped out of high school/not enrolled	1.49 (1.03 – 2.15)	.03	1.51 (1.06 – 2.16)	.02
Receiving public assistance	1.01 (.81 – 1.25)	.95	1.02 (.83 – 1.26)	.85
Biological mother less than 20 years old at first birth	1.13 (.94 – 1.35)	.20	1.12 (.93 – 1.34)	.23
Family Structure (ref: two parents)				
One biological parent only	1.10 (.91 – 1.32)	.33	1.09 (.90 – 1.31)	.38
Other	1.07 (.74 – 1.55)	.71	1.06 (.75 – 1.51)	.73
Mother's education less than high school	1.19 (.89 – 1.58)	.23	1.17 (.89 – 1.54)	.27
Parent's income (ref: < $15,000)				
$15,000 to $44,999	.95 (.73 – 1.23)	.70	.97 (.75 – 1.26)	.81
$45,000 to $74,999	1.12 (.83 – 1.51)	.45	1.13 (.84 – 1.52)	.42
$75,000 or greater	.92 (.67 – 1.28)	.64	.95 (.69 – 1.31)	.75
Don't know/Refused	.87 (.66 – 1.14)	.30	.87 (.66 – 1.13)	.30
Religious Importance	1.07 (.98 – 1.17)	.16	1.07 (.98 – 1.17)	.13
Age	1.01 (.87 – 1.18)	.89	1.03 (.89 – 1.20)	.71
**Personality**				
Extraversion	1.14 (.97 – 1.35)	.12	1.11 (.94 – 1.31)	.21
Agreeableness	.95 (.78 – 1.15)	.58	.94 (.77 – 1.15)	.54
Conscientiousness	.78 (.65 - .93)	.005	.79 (.66 - .94)	.007
Neuroticism	1.01 (.83 – 1.23)	.96	1.00 (.83 – 1.22)	.98
Intellect/Imagination	1.05 (.86 – 1.27)	.64	1.06 (.87 – 1.28)	.59
**Contact Information/Mode**				
Contact information: provided email and phone (ref: provided only one or no	.73 (.56 – .95)	.02	.73 (.57 – .94)	.01

contact information)				
Reminder mode: text and email (ref: only one or no mode for reminder)	1.02 (.86 – 1.22)	.81	1.01 (.85 – 1.20)	.91
**Adolescent Pregnancy-Related Experiences**				
Age at first sex 16 years or less	1.09 (.88 – 1.34)	.45	1.08 (.88 – 1.34)	.45
Number of sexual partners 2 or more	1.13 (.90 – 1.40)	.29	1.07 (.86 – 1.33)	.55
Ever had sex without contraception	1.23 (1.01 – 1.48)	.04	1.18 (.98 – 1.42)	.09
Number of pregnancies (ref: zero)				
One	1.15 (.91 – 1.47)	.25	1.10 (.87 – 1.40)	.42
Two or more	1.34 (.95 – 1.88)	.10	1.24 (.89 – 1.74)	.21
**Pregnancy-Related Changes since prior Journal**				
Sex (ref: no change)				
Stopped having sex			1.21 (1.08 – 1.35)	.001
Started having sex			1.17 (1.03 – 1.32)	.01
Partner transitions (ref: no change)				
Break-up (partner at time 1; no partner at time 2)			1.39 (1.21 – 1.61)	<.001
New partner (no partner at time 1; partner at time 2)			1.76 (1.53 – 2.03)	<.001
Partner switch (partner at time 1; different partner at time 2)			3.09 (2.56 – 3.74)	<.001
Contraceptive Use (ref: no change)				
Stopped using contraception			1.28 (1.10 – 1.49)	.001
Started using contraception			1.14 (.98 – 1.32)	.09
Pregnancy (ref: no change)				
Pregnancy ended			6.97 (5.39 – 9.01)	<.001
New pregnancy			5.57 (4.26 – 7.29)	<.001
Chi-square	1224.36		2396.64	
Degrees of freedom	28		37	
